# Hypoxia Promotes the Stemness of Mesangiogenic Progenitor Cells and Prevents Osteogenic but not Angiogenic Differentiation

**DOI:** 10.1007/s12015-024-10749-9

**Published:** 2024-06-24

**Authors:** Irene Sofia Burzi, Paolo Domenico Parchi, Serena Barachini, Eleonora Pardini, Gisella Sardo Infirri, Marina Montali, Iacopo Petrini

**Affiliations:** 1 Department of Translational Research and of New Surgical and Medical Technologies, University of Pisa, Via Savi 2, 56125 Pisa, Italy; 2 Department of Clinical and Experimental Medicine, University of Pisa, Via Roma 67, 56125 Pisa, Italy

**Keywords:** Hypoxia, Mesangiogenic progenitor cells, Stem cells, Mesenchymal stem cells, Angiogenic differentiation, Osteogenic differentiation

## Abstract

The stem cell niche in the bone marrow is a hypoxic environment, where the low oxygen tension preserves the pluripotency of stem cells. We have identified mesangiogenic progenitor cells (MPC) exhibiting angiogenic and mesenchymal differentiation capabilities in vitro. The effect of hypoxia on MPC has not been previously explored. In this study, MPCs were isolated from volunteers' bone marrow and cultured under both normoxic and hypoxic conditions (3% O2). MPCs maintained their characteristic morphology and surface marker expression (CD18 + CD31 + CD90-CD73-) under hypoxia. However, hypoxic conditions led to reduced MPC proliferation in primary cultures and hindered their differentiation into mesenchymal stem cells (MSCs) upon exposure to differentiative medium. First passage MSCs derived from MPC appeared unaffected by hypoxia, exhibiting no discernible differences in proliferative potential or cell cycle. However, hypoxia impeded the subsequent osteogenic differentiation of MSCs, as evidenced by decreased hydroxyapatite deposition. Conversely, hypoxia did not impact the angiogenic differentiation potential of MPCs, as demonstrated by spheroid-based assays revealing comparable angiogenic sprouting and tube-like formation capabilities under both hypoxic and normoxic conditions. These findings indicate that hypoxia preserves the stemness phenotype of MPCs, inhibits their differentiation into MSCs, and hampers their osteogenic maturation while leaving their angiogenic potential unaffected. Our study sheds light on the intricate effects of hypoxia on bone marrow-derived MPCs and their differentiation pathways.

**Graphical Abstract:**

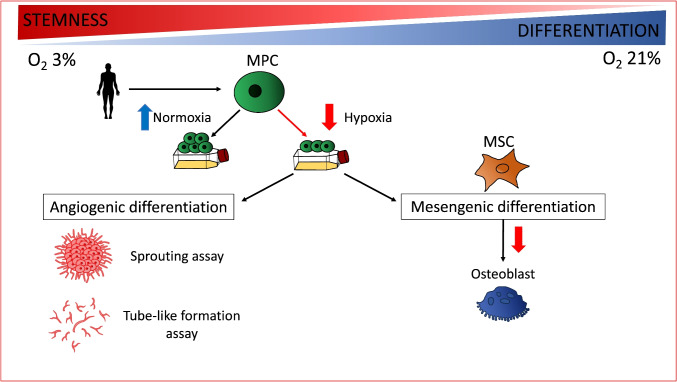

## Introduction

Adult bone marrow stem cells can differentiate into mature blood elements [1]. Moreover, they are well-documented to give rise to endothelial progenitor cells and mesenchymal stem cells (MSCs), which can further differentiate into osteogenic tissue, chondrocytes, and adipocytes [2]. In-vitro, MSC differentiation can be directed towards several cell types for example cardiomyocytes [2, 3].

We previously identified a cell population derived from human bone marrow exhibiting multipotent lineage progenitor characteristics [4]. These cells have demonstrated the capacity for differentiation into both mesenchymal and endothelial lineages under specific conditions. Hence, we have designated this cell population as mesangiogenic progenitor cells (MPC). MPCs grow in a medium enriched with human serum [5] and exhibit distinct morphological, phenotypic, and molecular features compared to MSCs: they are round, with a thick and highly refractive central region, and demonstrate strong adherence to plastic. Like stem cells, MPCs display a slow cycling nature and do not express Ki67. Compared to MSCs, MPCs have longer telomeres and express pluripotency-associated transcription factors NANOG and OCT4 [6]. We have demonstrated a hierarchical model of differentiation: MPCs can differentiate into either MSCs or endothelial precursor cells [7]. The process of differentiation advances through the commitment to an intermediate cell population, referred to as early MSCs. Through our investigation of WNT signaling activation during MPC differentiation, we have demonstrated the involvement of the non-canonical WNT5/calmodulin pathway in the commitment of MPCs to early MSCs [8]. It is interesting to note that inhibition of MSC differentiation by non-canonical WNT5/calmodulin signaling does not impact endothelial induction, confirming the specific involvement of the WNT5/calmodulin pathway in mesenchymal lineage differentiation [9]. Indeed, MPCs possess angiogenic potential, as evidenced by their ability to form spheroids and sprout when seeded in 3D Matrigel cultures. These cells express CD31 and NESTIN, akin to progenitor endothelial cells, but do not express CD146 and CD271, which are typical markers of pericytes. These findings suggest that MPCs may represent an early stem-like progenitor with the potential for angiogenesis [8, 10, 11].

Hypoxia maintains the pluripotent state of embryonic stem cells in the blastocyst and promotes stemness through somatic reprogramming induced by Yamanaka’s genes (OCT3/4, SOX2, KLF4, and MYC) [12]. Similarly, the perivascular niche of bone marrow is a hypoxic region, where the oxygen tension can drop to 9.9 mmHg [13]. This low oxygen concentration prevents the degradation of HIF-1α [14, 15]. In vitro, hypoxia maintains the stemness and pluripotency of human pluripotent stem cells by reducing spontaneous differentiation[16] and favors their self-renewal through the activation of the HIF pathway: HIF-2α and HIF-3α are upregulated and translocated from the cytoplasm to the nucleus, where they induce the expression of OCT4 and inhibit the transcription of HIF-1α, respectively [17]. Controversial data have been reported regarding MSCs, with some studies suggesting that hypoxic conditions stimulate proliferation and cell cycle progression, resulting in a significant increase in the G2/S/M population [15, 18, 19]. On the other hand, hypoxia markedly decreases the colony formation capacity of MSCs, suggesting an impaired self-renewal ability [20]. Others report that the stabilization of HIF-1α protein selectively enhances the colony-forming ability of MSCs but does not influence their overall proliferation [21].

The impact of hypoxia on MPCs has not been determined yet. Therefore, our aim is to evaluate the effect of hypoxic cultures on the self-renewal properties as well as the mesenchymal and angiogenic differentiation of MPCs. These findings could offer new insights into the role of hypoxia in regulating the differentiation of human pluripotent stem cells and MSCs.

## Materials and Methods

This trial was conducted in accordance with the Declaration of Helsinki and received approval from the Ethics Committee of the Tuscany Region for Clinical Trials—Section of the Northwest Area (CEAVNO): protocol number 1383/2015.

### Isolation of Mesangiogenic Progenitor Cells from Bone Marrow

We obtained bone marrow samples from subjects undergoing total hip replacement surgery, after obtaining written informed consent. Twenty milliliters of bone marrow were drawn into two 20-ml syringes containing 2500 units of heparin (Roche, Basel, Switzerland) and promptly transported to the cell culture facility. Upon arrival, the bone marrow was diluted with D-PBS (Gibco, Life Technologies Corporation, New York, NYC, USA) and carefully layered over Ficoll-Paque PREMIUM (GE Healthcare Bio-sciences, Uppsala, Sweden). Subsequently, it was centrifuged at 400 × g for 20 min at room temperature with the brake disabled.

MPCs were isolated from bone marrow mononuclear cells as previously described [22]. Briefly, the bone marrow mononuclear cells were harvested from the interface between the two phases and washed twice with fresh culture medium. The cells were then seeded at a density of 2.4–3.2 × 10^6^ cells per square centimeter in T-75 flasks for suspension cultures (Sarstedt, Nümbrecht, Germany) using low-glucose (1,000 mg/L) Dulbecco’s modified Eagle’s medium (DMEM, Gibco) supplemented with 10% pooled human AB-type serum (PhABS) of US origin (Sigma, Saint Louis, MO, USA), 5% Pen Strep (Gibco), and 5% GlutaMAX (Gibco) (DMEM + 10% PhABS). Non-adherent cells were removed after 48 h.

The cultures were maintained under both hypoxic (3% O_2_) and normoxic (20% O_2_) conditions in an ICO50 Memmert Incubator at 37 °C (Memmert GmbH + Co., Schwabach, Germany). After 7 days of culturing, MPCs were harvested using TrypLE Select (Life Technologies) and counted under a microscope after staining with Trypan Blue (Gibco), utilizing a Burker chamber. The cell count was repeated for 18 subjects, and the number of cells harvested under normoxic and hypoxic conditions was compared using a paired T-test in GraphPad Prism (GraphPad software v5, San Diego, CA, USA).

### Mesengenic Differentiation

Passage 1 (p1) MSC cultures were obtained from MPCs cultured under hypoxic conditions. After 7 days of culture in hypoxia, MPCs were detached using TrypLE Select (Gibco) for approximately 20 min. Following a wash with PBS, they were counted and seeded at a concentration of 3000–5000 cells per square centimeter in 6-well adhesive culture plates, then incubated in DMEM supplemented with 10% PhABS. After 24 h, the culture medium was replaced with StemMACS™ MSC Expansion Media Kit XF (Miltenyi Biotec, Bergisch Gladbach, Germany) to induce mesenchymal differentiation. Medium changes were performed twice a week for a total of 7 days of culture. Similarly, p1 MSCs were generated under normoxic conditions. Hypoxic and normoxic p1 MSC cultures were observed daily under a microscope to monitor survival and growth rate, and representative photographs were taken on days 3, 5, and 7.

### Flow Cytometry

The immunophenotype of MPCs and MSCs was evaluated using flow cytometry. MPCs cultured under normoxic, and hypoxic conditions for 7 days were collected and washed in PBS and MACSQuant™ Running Buffer (Miltenyi Biotech). Similarly, p1 MSCs derived from MPCs and grown for 3, 5, and 7 days in StemMACS were harvested and washed. Subsequently, cells were incubated for 15 min at 4 °C with the following fluorochrome-conjugated antibodies: anti-CD73 PE, anti-CD31 PE-Vio770, anti-CD18 APC, and anti-CD90 FITC (Miltenyi Biotech). The gating strategy included the selection of single-cell events on the FSC-A versus FSC-H plot and the selection of cellular events on the FSC-A versus SSC-A plot, followed by visualization of each fluorescence. Furthermore, CD18 versus CD31 plots were displayed to discriminate between MPCs (CD18 + CD31 + CD90-CD73-) and MSC differentiating cultures (CD18-CD31-CD90 + CD73 +). The experiment was conducted in triplicate with cells obtained from multiple subjects. Samples were acquired using the MACSQuant Flow Cytometer (Miltenyi Biotech) and analyzed using MACSQuantify Software (Miltenyi Biotech).

### Cell Cycle Assay

The cell cycle was analyzed by flow cytometry using propidium iodide (PI), a nucleic acid dye that binds to DNA and reveals its intracellular amount. Cells in the G2-M phase have double the DNA content compared to those in the G0-1 phase, while cells in the S phase have an intermediate amount.

MPCs were cultivated under hypoxic and normoxic conditions, and after 7 days, they were detached and reseeded at a concentration of 2 × 10^4^ cells per square centimeter in 6-well adherent culture plates. After 24 h of incubation, the culture medium DMEM + 10% PhABS was replaced with StemMACS MSC Expansion Media Kit XF to promote the differentiation of MPCs into p1 MSCs. After 5 and 7 days of culture under hypoxic differentiating conditions, the cells were detached using Trypsin and suspended in Sample Buffer (1 g of Glucose in 1 L of Phosphate-buffered saline (PBS) without Ca^2+^ or Mg^2+^). The suspension was filtered through a 0.22 µm filter, and the cells were washed twice by centrifugation at 300xg for 10 min at 4 °C. After aspirating the supernatant, the pellet was resuspended by vortexing in the residual buffer (approximately 0.1 mL/10^6^ cells), and 1 mL of ice-cold 70% ethanol was slowly added drop by drop. Fixation was performed overnight (> 18 h) at 4 °C. Subsequently, the cells were washed and incubated for 30–40 min at room temperature with 1 mL of staining solution: propidium iodide (50 µg/mL) (Miltenyi Biotech) and RNase A (100 Kunitz units/mL) (Qiagen, Hilden, Germany) in sample buffer. A total of 4 samples from multiple subjects were acquired using the MACSQuant® Flow Cytometer (Miltenyi Biotech) and analyzed with FlowJo v5 (FlowJo 10.9.0, BD Life Sciences, Franklin Lakes, NJ, USA). Statistical significance was assessed using Wilcoxon test for cells grown in normoxic and hypoxic conditions, utilizing GraphPad Prism (GraphPad Software v5).

### AlamarBlue Reduction Assay

AlamarBlue estimates cell proliferation in our cultures. It contains a REDOX indicator that changes color in response to the chemical reduction of the culture medium caused by cell growth.

The p1 MSCs cultured in 6-well tissue culture plates were incubated in 1.5 mL of culture media with 10% alamarBlue (Biorad Laboratories Inc., Hercules, CA, USA) on day 5 and day 7 of differentiation. The absorbance at 570 nm and 600 nm was measured 6 h and 24 h after the addition of alamarBlue to the culture using a Benchmark Plus microplate spectrophotometer (Biorad). The percentage reduction of alamarBlue was calculated according to the manufacturer's instructions. The test was performed on samples from 4 different subjects, and the percentage of reduced alamarBlue in the hypoxic and normoxic cultures was compared using Wilcoxon test in GraphPad Prism.

### Osteogenic Differentiation

MPCs were initially cultured under hypoxic conditions for 7 days using DMEM + 10% PhABS. Subsequently, they were detached, counted, and seeded to promote differentiation into p1 MSCs. Hypoxic p1 MSC cultures were continued for 8–10 days until they reached 80% confluence in the adherent culture plate. The p1 MSCs were then detached using trypsin and counted with Trypan Blue. Twenty-thousand cells per square centimeter were seeded in adherent culture plates, and StemMACS™ MSC Expansion Media Kit XF was added to obtain p2 MSC cultures. The cultures were maintained for 3–5 days until cells reached 80% confluence. The culture medium was then replaced with StemMACS OsteoDiff Media (Miltenyi Biotech) and replaced every three days. The differentiation cultures were continued for 10 days, after which Alizarin Red S (Sigma) staining was performed to visualize hydroxyapatite deposits. Briefly, the cells were washed with PBS at room temperature and fixed in ice-cold 70% ethanol for 1 h at 4 °C. After fixation, the cells were washed with distilled water and incubated for 10–15 min at room temperature with agitation in 40 mM pH 4.2 Alizarin Red S. Finally, they were washed with distilled water to remove excess dye, and hydroxyapatite deposits were visualized. Similarly, the assessment of osteogenic differentiation was performed on cells grown under normoxic conditions. Pictures were taken using an inverted fluorescence DM IRB Leica microscope (Leica Microsystems, Wetzlar, Germany), equipped with LAS image acquisition software (Leica Microsystems). Quantification of stained areas was performed using ImageJ (ImageJ 1.54d software, Wayne Rasband and contributors National Institute of Health, USA). The differences in the percentage of stained areas were evaluated in 3 samples from different subjects using a paired t-test.

### Assessment of Sprouting Angiogenesis in 3D Culture

Spheroids were created using the hanging drop technique as previously described [7]. Briefly, MPCs cultured under hypoxic conditions were collected and washed with PBS. After centrifugation, the cells were resuspended in DMEM + 10% PhABS to create drops containing 100,000 cells in 20 µL of culture medium. These drops were deposited on a Petri dish posed upside down in the incubator and maintained for 24–48 h. After the incubation period, the spheroids formed within the drops were harvested and placed on a thick layer of Geltrex LDEV-Free Reduced Growth Factor Basement Membrane Matrix (Life Technologies, Bleiswijk, Netherlands) and EGM-2 culture medium (Lonza, Walkersville, MD, USA) in a 48-well tissue culture plate. The spheroids were observed for a total of 7 days in both hypoxic and normoxic conditions, with the medium changed every 48 h. The presence of branches was monitored throughout the experiment by measuring the distance between the last invading cell and the spheroid's edge, as well as the quantification of branch density in representative sections. Measurements were independently performed by three operators, and mean values were recorded. The experiments were repeated 4 times each using different cells obtained from different subjects, and statistical significance was assessed using a paired Student’s t-test.

### Tube-like Formation Assay

We seeded MPC cells cultured under hypoxic and non-spheroid-inducing conditions on Geltrex in EGM-2 and maintained them in 3% hypoxia. Specifically, we seeded 50,000 cells per square centimeter of MPCs cultured under hypoxia for 7 days directly into a 48-well plate coated with a layer of Geltrex (Life Technologies). These cells were then maintained in EGM-2 culture medium (Lonza) for 72 h. Phase-contrast microphotographs were taken and processed for image analysis to measure tube lengths each day of the experiment. MPCs directly seeded on Geltrex and cultured under normoxic conditions served as negative controls. The experiments were repeated 4 times each using cells obtained from different subjects, and statistical significance was assessed using a paired Student’s t-test.

## Results

### Morphological Characterization of MPCs and Cytofluorimetric Evaluations

**Fig. 1:**
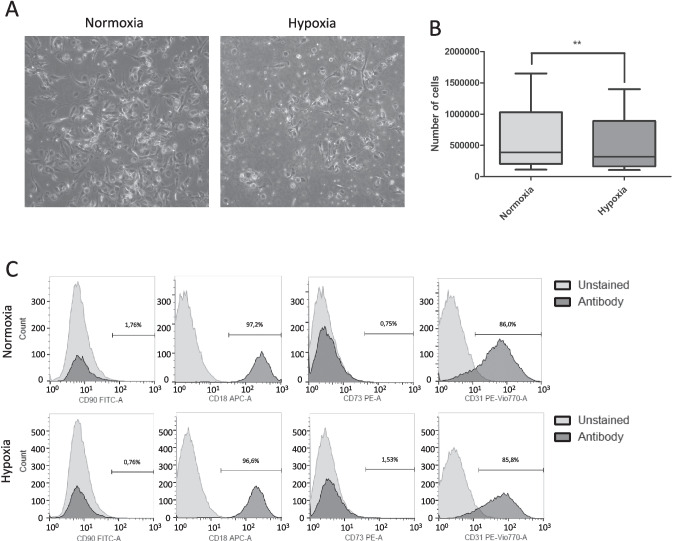
Mesangiogenic Progenitor Cells (MPCs) cultured under normoxic and hypoxic conditions. **A** MPCs cultured under hypoxic conditions show the same morphologic of normoxic MPCs, maintaining the characteristic fried egg-like shape. **B** The yield of MPCs after 7 days of culture in DMEM + 10% PhABS was significantly lower in hypoxic than in normoxic conditions (** = *p* < 0.01). **C** According to flow cytometric analysis, the cells expressed MPC surface markers (CD31 + CD18 +) and lacked mesenchymal stem cell (MSC) surface markers (CD73-CD90-) after 7 days of culture in DMEM + 10% PhABS, under both hypoxic and normoxic conditions. Overlay histograms show unstained samples (light gray) and antibody-stained samples (dark gray). The grey bars on the charts describe the percentage of cells positive to the marker. The FITC, fluorescein isothiocyanate; APC, allophycocyanin; PE, phycoerythrin; PE-Vio770, phycoerythrin and Vio®770

MPCs were isolated from the bone marrow of 18 subject who underwent surgery for hip replacement. MPCs maintained their characteristic fried-egg morphology in both hypoxic (3% O_2_) and normoxic conditions [23] (Fig. 1A). The number of MPCs collected after 7 days of culture was lower when incubated in hypoxic conditions compared to normoxic conditions (mean 506,478; standard deviation (SD) ± 411,125 vs. 619,011 SD ± 503,049; paired t-test p = 0.009) (Fig. 1B). Their MPC phenotype was confirmed by flow cytometry. Specifically, the cells collected after 7 days in hypoxic and normoxic conditions expressed CD31 + CD18 + but not CD90-CD73-: markers characteristic of MPC (Fig. 1C).

### Mesengenic Differentiation

**Fig. 2:**
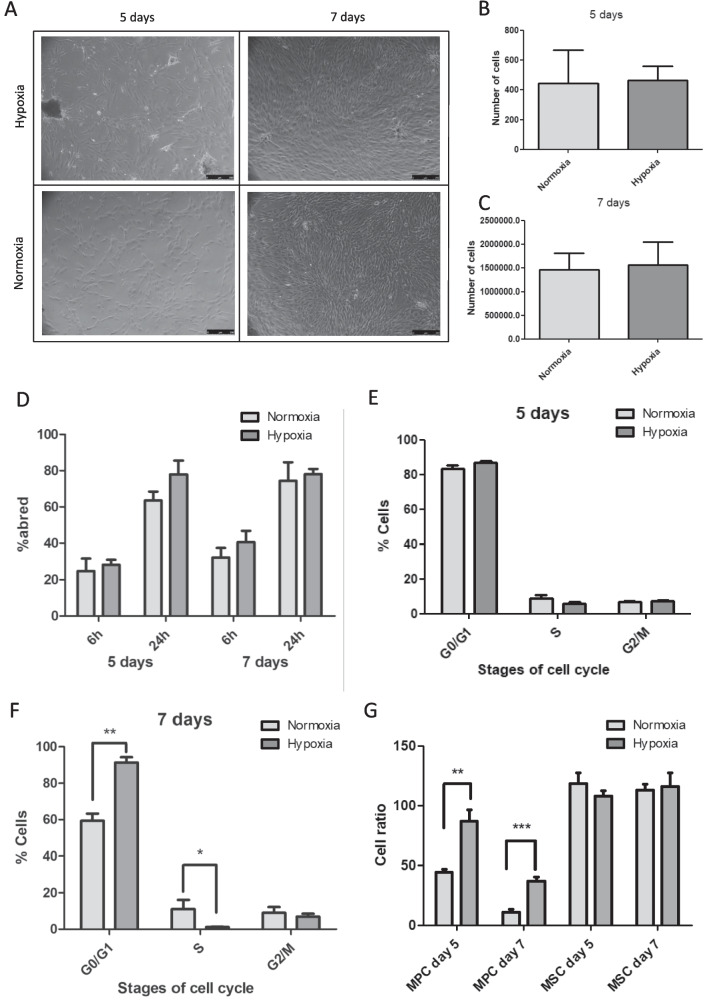
Mesenchymal differentiation of MPCs obtained from hypoxic cultures. **A** MPCs cultured under hypoxic conditions can differentiate into p1 MSC, adopting the characteristic fibroblastic spindle-like shape. **B** The cell count observed at 10X magnification under a microscope after 5 days of culture with StemMACS MSC Expansion Medium did not differ between hypoxic and normoxic conditions. **C** The cell yields after 7 days of culture with StemMACS MSC Expansion Medium did not differ between hypoxic and normoxic conditions. **D** The proliferative potential of cells cultured under hypoxic and normoxic conditions was assessed using the alamarBlue reduction test after 5 and 7 days of culture. Absorbance was measured after 6 and 24 h of incubation with alamarBlue. No significant differences were observed between hypoxic and normoxic conditions at any time point. (%abred = percentage of alamarBlue reduction). **E** Cell cycle analysis was conducted on cells cultured for 5 days following incubation with StemMACS MSC Expansion Media XF. No differences were observed in the three phases between hypoxic and normoxic conditions after 5 days of differentiation. **F** Cell cycle analysis was performed on cells cultured for 7 days following incubation with StemMACS MSC Expansion Media XF. After 7 days of differentiation, there were significantly more cells in the G0-G1 phase under hypoxic conditions, while an increased number of cells was observed in the S phase under normoxic conditions (** = *p* < 0.01; * = *p* < 0.05). **G** During MSC differentiation, the ratio of MPC percentages in hypoxic cultures on day 5 and day 3, as well as on day 7 and day 3, significantly differed from the ratio observed in normoxic cultures (** = *p* < 0.01; *** = *p* < 0.001)

MPCs were differentiated into p1 MSCs under hypoxic and normoxic conditions and reached confluency after 7 days when seeded in a 6-well plate (Fig. 2A). Considering that MPCs are quiescent while MSCs are proliferative, we evaluated the growth potential of p1 MSCs in hypoxic and normoxic conditions. After 5 days of culture, we counted the number of adherent cells present in a 10 × microscope field for 5 samples for both conditions. We observed an average of 442 cells (SD ± 223) and 461 cells (SD ± 97) in normoxic and hypoxic conditions, respectively (Wilcoxon p = 0.8) (Fig. 2B). The total number of cells harvested after 7 days of culture in MSC differentiating medium was comparable between hypoxic and normoxic conditions (mean number of cells = 1.5 × 10^6^ SD ± 0.98 × 10^6^ vs. 1.46 × 10^6^ SD ± 0.69 × 10^6^, paired t-test p = 0.59) (Fig. 2C).

Moreover, we assessed proliferation using alamarBlue by calculating the proportion of dye reduction after 6 and 24 h of incubation. After 5 days of culture, we did not observe differences between hypoxic and normoxic conditions. Specifically, after 6 h, the mean percentage of alamarBlue reduction was 78 (SD ± 15) and 64 (SD ± 10) in hypoxic and normoxic conditions, respectively (Wilcoxon p = 0.62). Similarly, after 24 h, the mean percentage of alamarBlue reduction was 28 (SD ± 6) and 25 (SD ± 13.9) in hypoxic and normoxic conditions, respectively (Wilcoxon p = 0.25). Following 7 days of culture, we still did not observe differences between hypoxic and normoxic conditions. After 6 h, the mean percentage of alamarBlue reduction was 41 (SD ± 12) and 32 (SD ± 10) in hypoxic and normoxic conditions, respectively (Wilcoxon p = 0.37). Likewise, after 24 h, the mean percentage of alamarBlue reduction was 78 (SD ± 6) and 74 (SD ± 21) in hypoxic and normoxic conditions, respectively (Wilcoxon p = 0.87) (Fig. 2D).

Cell cycle analysis was conducted after 5 and 7 days of culture for 3 samples. After 5 days of culture, the proportion of cells in the G0/G1 phase was 83% (SD ± 4) and 87% (SD ± 3) in hypoxic and normoxic conditions, respectively (Wilcoxon p = 0.12). The proportion of cells in the S phase was 9% (SD ± 4) and 6% (SD ± 2) in hypoxic and normoxic conditions, respectively (Wilcoxon p = 0.12), while the proportion of cells in the G2 phase was 7% (SD ± 1) in both hypoxic and normoxic conditions (Wilcoxon test p = 1) (Fig. 2E). After 7 days, cell cycle analysis performed by flow cytometry on p1 MSCs revealed a higher percentage of cells in the G0/G1 phase (91% SD 3 ± vs. 59% ± SD 5; Wilcoxon test p = 0.003) and a lower percentage of cells in the S phase in hypoxic cultures compared to normoxic cultures at 7 days of differentiation (1% SD ± 0.04 vs. 15% SD ± 6; Wilcoxon test p = 0.02) (Fig. 2F).

In hypoxic conditions, we observed a proportionally higher number of MPCs at 5 and 7 days (ratio between day 5 and day 3 = 87 SD ± 10 vs 45 SD ± 2; unpaired t-test p = 0.0018; ratio between day 7 and day 3 = 37 SD ± 3 vs 11 SD ± 2; unpaired t-test p = 0.0004), while no significant differences were observed for hypoxic and normoxic MSCs (ratio measured between day 5 and day 3: 108 SD ± 5 vs 118 SD ± 9; unpaired t-test p = 0.15; ratio measured between day 7 and day 3: 116 SD ± 11 vs 113 SD ± 5; unpaired t-test p = 0.7) (Fig. 2G).

### Osteogenic Differentiation

**Fig. 3:**
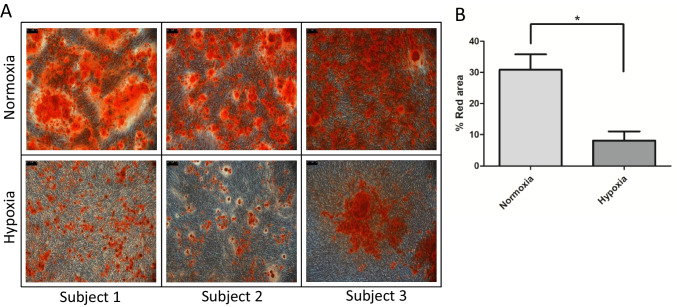
Osteogenic differentiation of MSCs obtained from hypoxic and normoxic cultures. **A** Cells differentiated in osteoblast were measured according to their production of hydroxyapatite crystals. Crystals were colored in red using Alizarin Red S staining. **B** Red areas were significantly larger in cultures grown in normoxic than in hypoxic conditions (* = *p* < 0.05)

P1 MSCs were differentiated into osteoblasts under both hypoxic and normoxic conditions. However, under hypoxic conditions, the differentiation process was slowed down, resulting in fewer hydroxyapatite deposits compared to normoxic cultures. Specifically, hydroxyapatite deposits, stained with Alizarin Red S, covered a larger proportion of the culture area in normoxic conditions (mean 31% SD ± 9) than in hypoxic cultures (8% SD ± 5; paired t-test p = 0.02) (Fig. 3A, 3B).

### Angiogenic Differentiation: Sprouting Assay and Tube-like Formation Assay

**Fig. 4:**
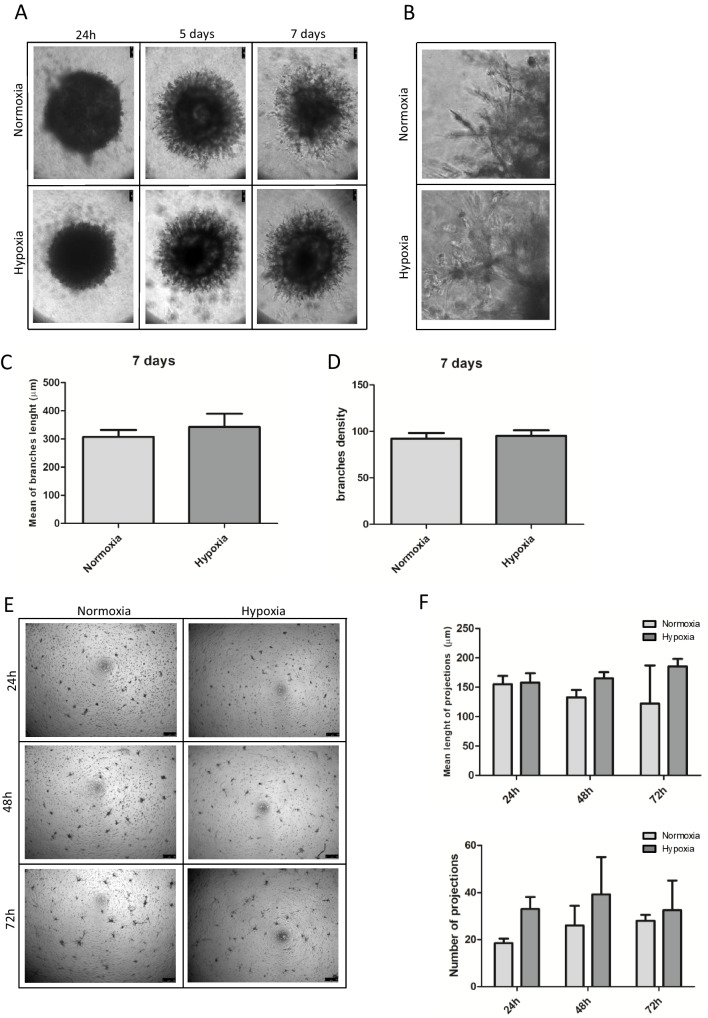
Angiogenic potential remain unaffected from hypoxic treatment. **A** Spheroids were formed from MPC cells grown in hypoxic and normoxic conditions. Sprouting of branches was observed when hypoxic and normoxic spheroids were placed on Geltrex matrix and incubated with EGM-2 media. Timepoints at 24 h, 5 and 7 days are shown for a spheroid grown in normoxic and hypoxic conditions. **B** Magnification of spheroids derived from normoxic and hypoxic MPCs at 7 days of sprouting assay. **C** The length of spheroid branches was evaluated under both normoxic and hypoxic conditions. No differences were observed in the average branch length between the two conditions. **D** The percentage of area occupied by spheroid branches, serving as an indicator of their density, was measured under both normoxic and hypoxic conditions, but no differences were observed. **E** MPC cells placed on Geltrex and grown in EGM-2 media did not form any tubular structure both in normoxic and hypoxic conditions. Cells projections were observed both in normoxic and hypoxic conditions. **F** The mean length of projections did not differ between cells grown in hypoxic and normoxic conditions when measurement after 24, 48 and 72 h of culture in EGM-2. Similarly, the number of projections counted in hypoxic and normoxic conditions did not differ after 24, 48 and 72 h of culture in EGM-2

We conducted two assays to evaluate angiogenic differentiation: the sprouting assay and the tube-like formation assay. Spheroids were generated from MPCs using the hanging drop technique and then cultured under hypoxic and normoxic conditions on a layer of Geltrex in EGM-2 culture medium for 7 days (Fig. 4A and B). Within 24 h, invading cells began to emerge from the edges of both hypoxic and normoxic MPC spheroids. Extended culture for 7 days revealed sprouting angiogenesis occurring in all directions from the hypoxic MPC spheroids, with distances from the edge estimated to range between 100 and 600 μm. We did not observe significant differences in the average length of branches between spheroids cultured under hypoxia and those cultured under normoxia (mean 342 μm SD ± 94 vs. 308 μm SD ± 49, paired t-test p = 0.35) (Fig. 4C). Furthermore, no significant differences were observed in the density of branches occupied by MPC spheroids cultured in hypoxia compared to those in normoxic conditions (mean hypoxic density 95 per square μm SD ± 12 vs mean normoxic density 92 per square μm SD ± 12; paired t-test p = 0.37) (Fig. 4D).

We had previously demonstrated that MPCs can form vascular structures only after the formation of sprouting spheroids, from which single cells can be dissociated and seeded onto a Geltrex layer to form tube-like structures[7]. Hence, we investigated whether hypoxia could enhance this phenomenon by directly seeding MPCs onto Geltrex without the intermediate step of spheroid formation. In the tube-like formation assay, MPCs cultured under hypoxic and normoxic conditions showed no significant differences in the appearance of vessel-like structures (Fig. 4E). Additionally, there were no significant differences in the average length and number of projections between MPCs seeded on Geltrex under hypoxic and normoxic conditions (average length of projections: 169 μm SD ± 14 and 137 μm SD ± 17; paired t-test p = 0.2; average number of projections: 35 SD ± 4 and 24 SD ± 5, paired t-test p = 0.07) (Fig. 4F).

## Discussion

Hypoxia preserves the stem cell characteristics of MPCs, inhibiting their differentiation into MSCs and subsequent osteogenic maturation. However, hypoxia does not appear to impact the angiogenic differentiation of MPCs.

In the bone marrow, hematopoietic stem cells reside in regions of severe hypoxia, with the lowest oxygen levels found in the deeper perisinusoidal and endosteal regions [13, 24]. Hypoxia contributes to the maintenance of the stem phenotype both in vivo and in vitro [25–28]. To assess the impact of hypoxia on the cells within the bone marrow niche, we examined its effect on MPCs and its influence on their differentiation. MPCs can be obtained from a unique sub-fraction of bone marrow mononuclear cells, named Pop#8. This population of stem cell is characterized by the expression of CD64^bright^, CD31^bright^, CD38 + , CD45^dim^ and the absence of CD14, CD34, CD66, CD3/20. Pop#8 has the ability to originate MPC in vitro when cultured in DMEM + 10% PhABS but lack the ability to generate MSC and spheroid when cultured in mesengenic condition or when they are induced to aggregate through the hanging drop technique. Hence, Pop#8 is the immediate precursor of MPCs [29]. While MSCs can originate from several tissues, MPCs have been isolated only from bone marrow [30, 31] using a standardized and reproducible procedure [22]. In vitro, MPCs exhibit stem-cell-like features, such as longer telomeres and the expression of pluripotency-associated transcription factors NANOG and OCT4. They also demonstrate a slower doubling time unless prompted to differentiate into rapidly growing MSCs. Compared to MSCs, MPCs lacked the CD73, CD90, and CD166 markers and expressed lower levels of CD105. However, they expressed integrins αL (CD11a), αM (CD11b), αX (CD11c), integrin β2 (CD18), and PECAM (CD31) [7]. MPCs are a homogeneous population of cells, whereas MSCs can be a mixture of elements at various stages of differentiation, which can sometimes hamper the reproducibility of experiments. MPCs remain stable for a long time and exhibit limited proliferative potential unless stimulated under differentiative conditions. MPCs retain pluripotent differentiative potential, capable of differentiating into both angiogenic and mesenchymal cells, which can further differentiate into adipocytes, chondrocytes, and osteoblasts. The stemness, angiogenic, and mesengenic potential of MPCs make them an attractive model for evaluating the effect of hypoxia on bone marrow stromal precursors.

In our experiments, hypoxia did not alter the morphology of MPCs, which remained rounded with a thick and highly refractive central region. Furthermore, hypoxia did not alter the expression of CD18 and CD31, which are characteristic markers of MPCs, while CD90 and CD73, markers of MSCs, remained negative. Hence, our method for MPC selection effectively prevents differentiation into MSCs under both normoxic and hypoxic conditions. Interestingly, the yield of MPCs isolated under hypoxic conditions was lower than that under normoxia, indicating a potential prolongation of their doubling time and a possible enhancement of their stemness phenotype. Notably, the effect of hypoxia on MPCs has not been previously reported in the literature. In contrast, the effect of hypoxia on MSCs remains controversial, with some studies suggesting increased expansion of MSCs [20, 32], whereas other suggest a reduced proliferation [33]. When MPCs were differentiated into early MSCs, we did not observe any difference in the total number of cells after 5 and 7 days between hypoxic and normoxic conditions. Moreover, there was no significant difference in the proliferation of p1 MSCs between hypoxic and normoxic conditions as measured by the alamarBlue assay. Similarly, no difference was observed in the cell cycle at 5 days. However, after 7 days of culture, we observed more cells in the G1/G0 phase and fewer cells in the S phase under hypoxic conditions. Since cells reached confluency after 7 days of culture in a 6-well plate, contact inhibition could explain these differences. Indeed, previous literature has described an increased proliferative potential of MSCs grown under hypoxic conditions, as well as the maintenance of the stemness phenotype of pluripotent stem cells [15, 18, 19]. Interestingly, the number of MPCs measured in p1 MSC culture was increased under hypoxic conditions at 5 and 7 days, whereas we did not observe a variation in the number of MSCs according to the oxygen concentration. This observation could be explained by an increased proliferative potential of MSCs in hypoxia, coupled with a reduced differentiation of MPCs into MSCs. Data suggest a differential effect of hypoxia on different stages of differentiation of bone marrow stem cells, with hypoxia maintaining MPCs in a more stem-like state and preventing their differentiation into MSCs. Like stem cells, MPCs are quiescent cells that exhibit slow proliferation unless they initiate their differentiation process [34]. Some reports indicate that HIF-1α can induce cell cycle arrest in the G0/G1 phase through the expression of p27 [35], and the overall activation of HIF-1α under hypoxic conditions promotes the undifferentiated state of human MSCs [20]. Hypoxia alters cellular metabolism, affecting mitochondrial respiration, reactive oxygen species (ROS) production, glycolysis, and fatty acid processing, resulting in a lower ATP energy yield. This may correlate with a reduction in cellular proliferation; however, the proliferative capacities of different cell types are variably affected by hypoxia [36]. MPCs exhibit a low proliferation rate, making it difficult to determine whether the reduction in proliferation induced by hypoxia is due to metabolic modifications or the preservation of the stemness phenotype. These two aspects are closely related, as the activation of the HIF-1α pathway and metabolic modifications both lead to reduced proliferation and the maintenance of a more stem-like phenotype. In contrast, hypoxia stimulates MSC proliferation, possibly through the activation of the PI3K/Akt pathway mediated by ROS.

MSCs retain the potential to differentiate into osteoblasts, adipocytes, and chondrocytes [23]. To assess the impact of hypoxia on the differentiation of MSCs derived from MPCs, we focused on osteogenic differentiation. Hypoxia affects lineage-specific differentiations of MSCs differently. For example, it promotes chondrogenic differentiation of MSCs partially through the HIF-1α pathway [37]. Contradictory results have been reported for adipogenic differentiation with some reports describing an enhancement and other a repression [38–40]. Whether hypoxia inhibits or promotes osteogenesis remains a topic of debate [41, 42]. In the literature, while some studies suggest that hypoxic environments can either maintain or even enhance multilineage differentiation [18, 33, 43], others indicate a reduced differentiation of MSCs cultured under low oxygen tensions [44, 45]. MSCs demonstrate elevated constitutive expression of HIF-1α mRNA and exhibit active glycolytic metabolism following isolation from bone marrow and umbilical blood. Following in vitro expansion and osteogenic differentiation, the mRNA level of HIF-1α and glycolytic activity decrease rapidly, while mitochondrial biogenesis increases, as indicated by elevated cell respiration and ROS generation [14]. Osteocytes reside in a region with low O2 tension in vivo (4–7%), but hypoxia attenuates osteogenic differentiation of MSCs. This could be due to hypoxia-dependent metabolism, which inhibits the metabolic switch toward oxidative phosphorylation metabolism: a process necessary for osteogenic differentiation of MSC [46]. We differentiated MSCs into osteoblasts under both hypoxic and normoxic conditions. Interestingly, in hypoxic conditions, we observed a decreased deposition of hydroxyapatite crystals, as visualized through Alizarin Red staining. Since osteoblasts dramatically reduce bone formation under hypoxic conditions [47], the indirect measurement of alizarin red could be affected. Only the direct measurement of osteoblast could overcome this limitation of our study. However, the literature suggests that in the bone marrow environment, the presence of HIF-1α inhibits the WNT pathway. This inhibition suppresses osteoblast proliferation and downregulates RUNX2 transcription in MSCs. RUNX2 is a crucial regulator of osteogenesis, and its suppression ultimately hinders MSC differentiation into osteoblasts [45, 48]. Our findings are consistent with this mechanism, as we observed a slower osteogenic differentiation in MSC cultures derived from MPCs under hypoxic conditions compared to normoxia. Additionally, our previous research indicated that WNT5 regulates the differentiation of MPCs into MSCs but does not impact the angiogenic differentiation of MPCs [9]. Reduced O2 tension in utero is necessary for the development of the cardiovascular-pulmonary system which originate from the growth plates of developing bones [12].

MPCs possess a unique ability to differentiate into vascular structures. In our in vitro experiments, we investigated the angiogenic potential of MPCs under both normoxic and hypoxic conditions. We found that angiogenic differentiation was not impaired by hypoxia. Indeed, we anticipate observing an enhancement of angiogenic differentiation under hypoxic conditions. In normoxic conditions or in tissues with oxygen tension above 5%, prolyl-4-hydroxylases (PHDs) hydroxylate specific proline residues of the HIF-1α protein [49, 50]. Following hydroxylation, HIF-1α undergoes ubiquitination by the ubiquitin ligase E3 von Hippel Lindau (VHL), leading to its subsequent degradation by the proteasome. Conversely, in hypoxic conditions, where oxygen tension falls below 5%, the hydroxylation of HIF-1α diminishes, leading to protein accumulation. This accumulated protein then translocates from the cytoplasm to the nucleus, where it forms a dimer with HIF-1β. This complex binds to hypoxia-responsive elements, recruits transcriptional coactivators, and triggers the expression of target genes, including VEGF, thereby promoting angiogenesis [51, 52]. However, in our experiments, we did not observe an increase in angiogenic potential under hypoxic conditions. One possible explanation could be that the angiogenic differentiation of MPC was already maximally stimulated by EGM-2, potentially masking any additional effect of hypoxia. Moreover, we did not observe spheroid sprouting when cultured in RPMI1640 on Geltrex, even when incubated under hypoxic conditions (data not shown). The angiogenic differentiation of MPC relies on the formation of spheroids, as MPC placed on Geltrex and stimulated with EGM-2 do not form tubular structures resembling vessels. Hypoxia alone does not induce MPC to form vascular structures on Geltrex unless spheroids have formed. Therefore, spheroid formation remains a necessary step to guide MPC differentiation towards angiogenesis, even under hypoxic conditions. Interestingly, the MPC angiogenic fate is not suppressed by the inhibition of the WNT pathway [9]. In multiple myeloma, MSC differentiation is inhibited while angiogenic differentiation is preserved [53]. Thus, our results suggest that the angiogenic potential of MPC may not be further enhanced by hypoxia. However, we can speculate that there may be an overall increase in angiogenic potential in hypoxic conditions. If the differentiation of MPC into MSC is reduced, more pluripotent cells remain available for differentiation into endothelial progenitors.

We can hypothesize a hierarchical model of differentiation in which MPCs carry angiogenic potential and serve as precursors to MSCs, which can further differentiate into osteoblasts, chondrocytes, and adipocytes. Hypoxia affects these differentiation steps differently. It maintains MPCs in a more quiescent and stem-like state, whereas it is ineffective or may even promote the proliferation of MSCs. We observed reduced differentiation of MSCs into osteoblasts under hypoxic conditions. On the contrary an increased differentiation into chondrocytes have been reported in the literature [37]. Hypoxia does not directly affect the angiogenic capability of MPCs, suggesting that this property is already present in MPCs.

## Data Availability

Not applicable.

## References

[1] Mitchell, E., Spencer Chapman, M., Williams, N., Dawson, K. J., Mende, N., Calderbank, E. F., et al. (2022). Clonal dynamics of haematopoiesis across the human lifespan. *Nature,**606*(7913), 343–350. 35650442 10.1038/s41586-022-04786-y PMC9177428

[2] Jiang, Y., Jahagirdar, B. N., Reinhardt, R. L., Schwartz, R. E., Keene, C. D., Ortiz-Gonzalez, X. R., et al. (2002). Pluripotency of mesenchymal stem cells derived from adult marrow. *Nature,**418*(6893), 41–49. 12077603 10.1038/nature00870

[3] Orlic, D., Kajstura, J., Chimenti, S., Jakoniuk, I., Anderson, S. M., Li, B., et al. (2001). Bone marrow cells regenerate infarcted myocardium. *Nature,**410*(6829), 701–705. 11287958 10.1038/35070587

[4] Trombi, L., Pacini, S., Montali, M., Fazzi, R., Chiellini, F., Ikehara, S., et al. (2009). Selective culture of mesodermal progenitor cells. *Stem Cells and Development,**18*(8), 1227–1234. 19331526 10.1089/scd.2009.0054

[5] Montali, M., Barachini, S., Panvini, F. M., Carnicelli, V., Fulceri, F., Petrini, I., et al. (2016). Growth Factor Content in Human Sera Affects the Isolation of Mesangiogenic Progenitor Cells (MPCs) from Human Bone Marrow. *Frontiers in Cell and Developmental Biology,**4*, 114. 27800477 10.3389/fcell.2016.00114 PMC5065953

[6] Pacini, S., Carnicelli, V., Trombi, L., Montali, M., Fazzi, R., Lazzarini, E., et al. (2010). Constitutive expression of pluripotency-associated genes in mesodermal progenitor cells (MPCs). *PLoS ONE,**5*(3), e9861. 20360837 10.1371/journal.pone.0009861 PMC2845604

[7] Montali, M., Panvini, F. M., Barachini, S., Ronca, F., Carnicelli, V., Mazzoni, S., et al. (2017). Human adult mesangiogenic progenitor cells reveal an early angiogenic potential, which is lost after mesengenic differentiation. *Stem Cell Research & Therapy,**8*(1), 106. 28464921 10.1186/s13287-017-0562-x PMC5414340

[8] Pacini, S., & Petrini, I. (2014). Are MSCs angiogenic cells? New insights on human nestin-positive bone marrow-derived multipotent cells. *Frontiers in Cell and Developmental Biology,**2*, 20. 25364727 10.3389/fcell.2014.00020 PMC4207020

[9] Fazzi, R., Pacini, S., Carnicelli, V., Trombi, L., Montali, M., Lazzarini, E., et al. (2011). Mesodermal progenitor cells (MPCs) differentiate into mesenchymal stromal cells (MSCs) by activation of Wnt5/calmodulin signalling pathway. *PLoS ONE,**6*(9), e25600. 21980498 10.1371/journal.pone.0025600 PMC3183072

[10] Barachini S, Ghelardoni S, Madonna R. Vascular Progenitor Cells: From Cancer to Tissue Repair. J Clin Med. 2023;12(6). 10.3390/jcm12062399 PMC10059009 36983398

[11] Pacini, S., Fazzi, R., Montali, M., Carnicelli, V., Lazzarini, E., & Petrini, M. (2013). Specific integrin expression is associated with podosome-like structures on mesodermal progenitor cells. *Stem Cells and Development,**22*(12), 1830–1838. 23379672 10.1089/scd.2012.0423

[12] Simon, M. C., & Keith, B. (2008). The role of oxygen availability in embryonic development and stem cell function. *Nature Reviews Molecular Cell Biology,**9*(4), 285–296. 18285802 10.1038/nrm2354 PMC2876333

[13] Spencer, J. A., Ferraro, F., Roussakis, E., Klein, A., Wu, J., Runnels, J. M., et al. (2014). Direct measurement of local oxygen concentration in the bone marrow of live animals. *Nature,**508*(7495), 269–273. 24590072 10.1038/nature13034 PMC3984353

[14] Palomäki, S., Pietilä, M., Laitinen, S., Pesälä, J., Sormunen, R., Lehenkari, P., et al. (2013). HIF-1α is upregulated in human mesenchymal stem cells. *Stem Cells.,**31*(9), 1902–1909. 23744828 10.1002/stem.1435

[15] Estrada, J. C., Albo, C., Benguría, A., Dopazo, A., López-Romero, P., Carrera-Quintanar, L., et al. (2012). Culture of human mesenchymal stem cells at low oxygen tension improves growth and genetic stability by activating glycolysis. *Cell Death and Differentiation,**19*(5), 743–755. 22139129 10.1038/cdd.2011.172 PMC3321628

[16] Ezashi, T., Das, P., & Roberts, R. M. (2005). Low O2 tensions and the prevention of differentiation of hES cells. *Proceedings of the National Academy of Sciences of the United States of America,**102*(13), 4783–4788. 15772165 10.1073/pnas.0501283102 PMC554750

[17] Forristal, C. E., Wright, K. L., Hanley, N. A., Oreffo, R. O., & Houghton, F. D. (2010). Hypoxia inducible factors regulate pluripotency and proliferation in human embryonic stem cells cultured at reduced oxygen tensions. *Reproduction,**139*(1), 85–97. 19755485 10.1530/REP-09-0300 PMC2791494

[18] Grayson, W. L., Zhao, F., Bunnell, B., & Ma, T. (2007). Hypoxia enhances proliferation and tissue formation of human mesenchymal stem cells. *Biochemical and Biophysical Research Communications,**358*(3), 948–953. 17521616 10.1016/j.bbrc.2007.05.054

[19] Martin-Rendon, E., Hale, S. J., Ryan, D., Baban, D., Forde, S. P., Roubelakis, M., et al. (2007). Transcriptional profiling of human cord blood CD133+ and cultured bone marrow mesenchymal stem cells in response to hypoxia. *Stem Cells.,**25*(4), 1003–1012. 17185612 10.1634/stemcells.2006-0398

[20] Basciano, L., Nemos, C., Foliguet, B., de Isla, N., de Carvalho, M., Tran, N., et al. (2011). Long term culture of mesenchymal stem cells in hypoxia promotes a genetic program maintaining their undifferentiated and multipotent status. *BMC Cell Biology,**12*, 12. 21450070 10.1186/1471-2121-12-12 PMC3073900

[21] Park, I. H., Kim, K. H., Choi, H. K., Shim, J. S., Whang, S. Y., Hahn, S. J., et al. (2013). Constitutive stabilization of hypoxia-inducible factor alpha selectively promotes the self-renewal of mesenchymal progenitors and maintains mesenchymal stromal cells in an undifferentiated state. *Experimental & Molecular Medicine,**45*(9), e44. 24071737 10.1038/emm.2013.87 PMC3789268

[22] Montali M, Barachini S, Pacini S, Panvini FM, Petrini M. Isolating Mesangiogenic Progenitor Cells (MPCs) from Human Bone Marrow. J Vis Exp. 2016(113). 10.3791/54225 27500428

[23] Petrini, M., Pacini, S., Trombi, L., Fazzi, R., Montali, M., Ikehara, S., et al. (2009). Identification and purification of mesodermal progenitor cells from human adult bone marrow. *Stem Cells and Development,**18*(6), 857–866. 18991503 10.1089/scd.2008.0291 PMC3085824

[24] Parmar, K., Mauch, P., Vergilio, J. A., Sackstein, R., & Down, J. D. (2007). Distribution of hematopoietic stem cells in the bone marrow according to regional hypoxia. *Proceedings of the National Academy of Sciences of the United States of America,**104*(13), 5431–5436. 17374716 10.1073/pnas.0701152104 PMC1838452

[25] Cipolleschi, M. G., Dello Sbarba, P., & Olivotto, M. (1993). The role of hypoxia in the maintenance of hematopoietic stem cells. *Blood,**82*(7), 2031–2037. 8104535

[26] Ivanovic, Z., Hermitte, F., Brunet de la Grange, P., Dazey, B., Belloc, F., Lacombe, F., et al. (2004). Simultaneous maintenance of human cord blood SCID-repopulating cells and expansion of committed progenitors at low O2 concentration (3%). Stem Cells. 22(5):716–24. 10.1634/stemcells.22-5-716 15342936

[27] Hermitte, F., Brunet de la Grange, P., Belloc, F., Praloran, V., Ivanovic, Z. (2006). Very low O2 concentration (0.1%) favors G0 return of dividing CD34+ cells. Stem Cells. 24(1):65–73. 10.1634/stemcells.2004-0351 16123391

[28] Shima, H., Takubo, K., Tago, N., Iwasaki, H., Arai, F., Takahashi, T., et al. (2010). Acquisition of G₀ state by CD34-positive cord blood cells after bone marrow transplantation. *Experimental Hematology,**38*(12), 1231–1240. 20800645 10.1016/j.exphem.2010.08.004

[29] Pacini, S., Barachini, S., Montali, M., Carnicelli, V., Fazzi, R., Parchi, P., et al. (2016). Mesangiogenic Progenitor Cells Derived from One Novel CD64(bright)CD31(bright)CD14(neg) Population in Human Adult Bone Marrow. *Stem Cells and Development,**25*(9), 661–673. 26975798 10.1089/scd.2015.0344 PMC4854213

[30] Barachini, S., Pacini, S., Montali, M., Panvini, F.M., Carnicelli, V., Piolanti, N., et al. (2020). Mesangiogenic Progenitor Cells and musculoskeletal tissue regeneration: differences between adipose-derived and bone marrow-derived cells? J Biol Regul Homeost Agents. 34(5 Suppl. 1):33–8. IORS Special Issue on Orthopedics. 33739002

[31] Barachini, S., Montali, M., Panvini, F. M., Carnicelli, V., Gatti, G. L., Piolanti, N., et al. (2021). Mesangiogenic Progenitor Cells Are Tissue Specific and Cannot Be Isolated From Adipose Tissue or Umbilical Cord Blood. *Frontiers in Cell and Developmental Biology,**9*, 669381. 34291045 10.3389/fcell.2021.669381 PMC8287027

[32] Weijers, E. M., Van Den Broek, L. J., Waaijman, T., Van Hinsbergh, V. W., Gibbs, S., & Koolwijk, P. (2011). The influence of hypoxia and fibrinogen variants on the expansion and differentiation of adipose tissue-derived mesenchymal stem cells. *Tissue Engineering Part A,**17*(21–22), 2675–2685. 21830936 10.1089/ten.tea.2010.0661

[33] Holzwarth, C., Vaegler, M., Gieseke, F., Pfister, S. M., Handgretinger, R., Kerst, G., et al. (2010). Low physiologic oxygen tensions reduce proliferation and differentiation of human multipotent mesenchymal stromal cells. *BMC Cell Biology,**11*, 11. 20109207 10.1186/1471-2121-11-11 PMC2827377

[34] Liu, L., Michowski, W., Kolodziejczyk, A., & Sicinski, P. (2019). The cell cycle in stem cell proliferation, pluripotency and differentiation. *Nature Cell Biology,**21*(9), 1060–1067. 31481793 10.1038/s41556-019-0384-4 PMC7065707

[35] Kumar, S., & Vaidya, M. (2016). Hypoxia inhibits mesenchymal stem cell proliferation through HIF1α-dependent regulation of P27. *Molecular and Cellular Biochemistry,**415*(1–2), 29–38. 26920732 10.1007/s11010-016-2674-5

[36] Hubbi, M. E., & Semenza, G. L. (2015). Regulation of cell proliferation by hypoxia-inducible factors. *American Journal of Physiology. Cell Physiology,**309*(12), C775–C782. 26491052 10.1152/ajpcell.00279.2015 PMC4683214

[37] Kanichai, M., Ferguson, D., Prendergast, P. J., & Campbell, V. A. (2008). Hypoxia promotes chondrogenesis in rat mesenchymal stem cells: A role for AKT and hypoxia-inducible factor (HIF)-1alpha. *Journal of Cellular Physiology,**216*(3), 708–715. 18366089 10.1002/jcp.21446

[38] Fink, T., Abildtrup, L., Fogd, K., Abdallah, B. M., Kassem, M., Ebbesen, P., et al. (2004). Induction of adipocyte-like phenotype in human mesenchymal stem cells by hypoxia. *Stem Cells.,**22*(7), 1346–1355. 15579652 10.1634/stemcells.2004-0038

[39] Jiang, C., Sun, J., Dai, Y., Cao, P., Zhang, L., Peng, S., et al. (2015). HIF-1A and C/EBPs transcriptionally regulate adipogenic differentiation of bone marrow-derived MSCs in hypoxia. *Stem Cell Research & Therapy,**6*(1), 21. 25889814 10.1186/s13287-015-0014-4 PMC4559195

[40] Lin, Q., Lee, Y. J., & Yun, Z. (2006). Differentiation arrest by hypoxia. *Journal of Biological Chemistry,**281*(41), 30678–30683. 16926163 10.1074/jbc.C600120200

[41] Wagegg, M., Gaber, T., Lohanatha, F. L., Hahne, M., Strehl, C., Fangradt, M., et al. (2012). Hypoxia promotes osteogenesis but suppresses adipogenesis of human mesenchymal stromal cells in a hypoxia-inducible factor-1 dependent manner. *PLoS ONE,**7*(9), e46483. 23029528 10.1371/journal.pone.0046483 PMC3459928

[42] Xu, N., Liu, H., Qu, F., Fan, J., Mao, K., Yin, Y., et al. (2013). Hypoxia inhibits the differentiation of mesenchymal stem cells into osteoblasts by activation of Notch signaling. *Experimental and Molecular Pathology,**94*(1), 33–39. 22964414 10.1016/j.yexmp.2012.08.003

[43] Valorani, M. G., Montelatici, E., Germani, A., Biddle, A., D’Alessandro, D., Strollo, R., et al. (2012). Pre-culturing human adipose tissue mesenchymal stem cells under hypoxia increases their adipogenic and osteogenic differentiation potentials. *Cell Proliferation,**45*(3), 225–238. 22507457 10.1111/j.1365-2184.2012.00817.x PMC6622217

[44] Hung, S. P., Ho, J. H., Shih, Y. R., Lo, T., & Lee, O. K. (2012). Hypoxia promotes proliferation and osteogenic differentiation potentials of human mesenchymal stem cells. *Journal of Orthopaedic Research,**30*(2), 260–266. 21809383 10.1002/jor.21517

[45] Yang, D. C., Yang, M. H., Tsai, C. C., Huang, T. F., Chen, Y. H., & Hung, S. C. (2011). Hypoxia inhibits osteogenesis in human mesenchymal stem cells through direct regulation of RUNX2 by TWIST. *PLoS ONE,**6*(9), e23965. 21931630 10.1371/journal.pone.0023965 PMC3170288

[46] Hsu, S. H., Chen, C. T., & Wei, Y. H. (2013). Inhibitory effects of hypoxia on metabolic switch and osteogenic differentiation of human mesenchymal stem cells. *Stem Cells.,**31*(12), 2779–2788. 23733376 10.1002/stem.1441

[47] Utting, J. C., Robins, S. P., Brandao-Burch, A., Orriss, I. R., Behar, J., & Arnett, T. R. (2006). Hypoxia inhibits the growth, differentiation and bone-forming capacity of rat osteoblasts. *Experimental Cell Research,**312*(10), 1693–1702. 16529738 10.1016/j.yexcr.2006.02.007

[48] Chen, D., Li, Y., Zhou, Z., Xing, Y., Zhong, Y., Zou, X., et al. (2012). Synergistic inhibition of Wnt pathway by HIF-1α and osteoblast-specific transcription factor osterix (Osx) in osteoblasts. *PLoS ONE,**7*(12), e52948. 23300831 10.1371/journal.pone.0052948 PMC3531395

[49] Jaakkola, P., Mole, D. R., Tian, Y. M., Wilson, M. I., Gielbert, J., Gaskell, S. J., et al. (2001). Targeting of HIF-alpha to the von Hippel-Lindau ubiquitylation complex by O2-regulated prolyl hydroxylation. *Science,**292*(5516), 468–472. 11292861 10.1126/science.1059796

[50] Ivan, M., Kondo, K., Yang, H., Kim, W., Valiando, J., Ohh, M., et al. (2001). HIFalpha targeted for VHL-mediated destruction by proline hydroxylation: Implications for O2 sensing. *Science,**292*(5516), 464–468. 11292862 10.1126/science.1059817

[51] Shweiki, D., Itin, A., Soffer, D., & Keshet, E. (1992). Vascular endothelial growth factor induced by hypoxia may mediate hypoxia-initiated angiogenesis. *Nature,**359*(6398), 843–845. 1279431 10.1038/359843a0

[52] Tsuzuki, Y., Fukumura, D., Oosthuyse, B., Koike, C., Carmeliet, P., & Jain, R. K. (2000). Vascular endothelial growth factor (VEGF) modulation by targeting hypoxia-inducible factor-1alpha–> hypoxia response element–> VEGF cascade differentially regulates vascular response and growth rate in tumors. *Cancer Research,**60*(22), 6248–6252. 11103778

[53] Pacini, S., Montali, M., Mazziotta, F., Schifone, C. P., Macchia, L., Carnicelli, V., et al. (2019). Mesangiogenic progenitor cells are forced toward the angiogenic fate, in multiple myeloma. *Oncotarget,**10*(63), 6781–6790. 31827721 10.18632/oncotarget.27285 PMC6887577

